# Feasibility of newborn hearing screening in a public hospital setting in South Africa: A pilot study

**DOI:** 10.4102/sajcd.v63i1.150

**Published:** 2016-07-21

**Authors:** Amisha Kanji, Katijah Khoza-Shangase

**Affiliations:** 1 Department of Speech Pathology and Audiology, University of the Witwatersrand, South Africa

## Abstract

**Objectives:**

The current pilot study aimed to explore the feasibility of newborn hearing screening (NHS) in a hospital setting with clinical significance for the implementation of NHS. Context-specific objectives included determining the average time required to screen each neonate or infant; the most suitable time for initial hearing screening in the wards; as well as the ambient noise levels in the wards and at the neonatal follow-up clinic where screening would be conducted.

**Method:**

A descriptive, longitudinal, repeated measures, within-subjects design was employed. The pilot study comprised 11 participants who underwent hearing screening. Data were analysed using descriptive statistics.

**Results:**

The average time taken to conduct hearing screening using otoacoustic emissions and automated auditory brainstem response was 18.4 minutes, with transient evoked otoacoustic emissions taking the least time. Ambient noise levels differed between wards and clinics with the sound level readings ranging between 50 dBA and 70 dBA. The most suitable screening time was found to be the afternoons, after feeding times.

**Conclusion:**

Findings highlight important considerations when embarking on larger scale NHS studies or when planning a hospital NHS programme. Current findings suggest that NHS can be efficiently and effectively conducted in public sector hospitals in South Africa, provided that test time is considered in addition to sensitivity and specificity when deciding on a screening protocol; bar recognised personnel challenges.

## Introduction

Early detection of hearing loss is the initial stage to any Early Hearing Detection and Intervention (EHDI) programme and is conducted by means of newborn hearing screening (NHS). The implementation of NHS programmes has evolved over the years, particularly in developed countries, with universal newborn hearing screening (UNHS) being the recommended and most widely practiced approach in these contexts. However, NHS programmes in South Africa have not been standardised, nor uniformly implemented nationally, with documented differences between the public and private healthcare sectors. These differences in the approach to screening, screening protocols and the overall lack of NHS services in South Africa have all been documented in national surveys (Meyer & Swanepoel, 2011; Theunissen & Swanepoel, 2008).

A variety of objective screening measures may be employed within a NHS programme. These include otoacoustic emissions (OAEs), automated auditory brainstem response (AABR) and a combination of OAE and AABR (Berninger & Westling, 2011). Whilst OAEs are simple, fast and cheaper, they provide limited assessment of the auditory system. OAEs are also negatively impacted by vernix, middle ear fluid and ambient noise (Choo & Meinzen-Derr, 2010). In contrast, the AABR provides more information regarding the auditory system and provides better detection of auditory neuropathy in infants. However, the AABR requires more knowledge and expertise to conduct, which limits the number of screening personnel who can utilise it. It is also typically more costly and requires a longer test time to conduct (Choo & Meinzen-Derr, 2010) when compared to OAEs. These are all factors that may influence the implementation of a comprehensive screening programme in different contexts – particularly in resource constrained contexts such as South Africa. Limited studies have documented the test time during screening that has excluded preparation time. The entire screening activity (from start to finish) is what defines the success of a programme and is important when positioning notions of feasibility. The technical focus on the time related to the test procedure is also not aligned with models of clinical feasibility studies and is especially not aligned with the current study context (public healthcare) as is the grander intention of this pilot study. Cebulla and Shehata-Dieler (2012) concluded that conducting AABR screening, using the MB11 BERAphone, reduces test time when compared to other portable screening equipment.

Various EHDI position statements recommend the use of different screening measures for different screening contexts. The Joint Committee on Infant Hearing (JCIH), for example, recommends the use of OAE or AABR for infants admitted to well-infant nurseries and AABR for infants admitted to the neonatal intensive care unit (NICU) (JCIH, 2007). The HPCSA (2007) position statement, on the other hand, recommends the use of AABR for infants admitted to the NICU and OAE for screening during immunisation visits at primary healthcare clinics within the South African context. Review of the literature, however, indicates that the ideal hearing screening measure is yet to be defined (Guastini *et al*., 2010), with various protocols currently being used in different contexts. Such diverse recommendations call for further exploration and definition of feasible and context-specific screening protocols. Such exploration would have to include deliberating on the entire EHDI process, including follow-up as well as diagnostic or interventional components of EHDI. Aspects such as coverage rates, referral rates, recording and tracking systems, as well as follow-up rates, are crucial in such feasibility studies.

The feasibility of screening protocols may also include consideration of ambient noise levels within the screening environment, particularly as ambient noise levels may influence OAE screening outcomes. Presence of undetected and unmonitored high ambient noise levels may result in higher referral rates or false-positive rates (Olusanya, 2010), which not only influences the efficiency of the NHS programme but has cost implications for the programme. In addition, it can cause unnecessary emotional distress for parents due to false-positive hearing screening results (Poulakis, Barker & Wake, 2003).

There are a number of studies that have explored the feasibility of NHS. Some of these studies have focused on coverage rates, referral rates and tracking systems associated with UNHS (Ng, Hui, Lam, Goh & Yeung, 2004; Pisacane *et al*., 2013). Other studies have focused on the feasibility in terms of the type of screening protocol employed (Kumar *et al*., 2014; Qi *et al*., 2013). Feasibility studies are commonly performed in many clinical areas. These studies usually commence with some small-scale investigation or pilot study to determine the feasibility of conducting a larger scale study. Pilot studies also assist in assessing feasibility in terms of the process, resources, management and scientific aspects such as treatment efficacy (Thabane *et al*., 2010). The current pilot study focused on the process involved with screening procedures and logistical aspects as it aimed at exploring the feasibility of NHS in a public hospital setting, thereby assisting in guiding a larger scale study.

The South African public healthcare setting faces significant challenges with regard to numerous factors including burden of disease, limited resources, issues of linguistic and cultural diversity influencing the healthcare provision, socio-economic status of the population served etc. All these factors have been documented to play a major role in the provision of public healthcare throughout all levels of care, which includes audiology services. It is within this public health sector context that the current study was located.

## Methods

### Objectives of the pilot study

- Determine the average time required to screen each neonate or infant.
- Determine the most suitable time for the initial hearing screening in the wards.
- Determine the ambient noise levels in the wards and at the neonatal follow-up clinic.

The time taken to conduct the hearing screening is critical when screening services are planned in any environment, but more so in an under-resourced environment where personnel:infant ratio is unfavourable and time limitations exist, irrespective of whether the screening personnel consists of a highly skilled audiologist, speech therapist, trained nursing staff or volunteers. Within the South African context, the general shortage of healthcare personnel, with a high demand to capacity ratio for audiologists, poses a significant challenge for feasibility of implementing NHS. Furthermore, the fact that there are still no promulgated minimum standards of training for professionals other than audiologists to conduct NHS creates further challenges. It is for these reasons that it becomes important to establish normative data around time taken to conduct screening as this evidence allows for approximations of how many newborns or infants can reasonably and accurately be screened within a given time period.

The second objective of the study was to ensure that the hearing screening did not disrupt other nursing duties and/or ward rounds. Determining the appropriate time for hearing screening could also inform the researcher as to possible times at which caregivers would be present in the wards in order to obtain informed consent. Such information is crucial for the sustainability of a NHS programme and for ethical practice. It would allow for efficient information counselling when caregivers are present. By identifying suitable times for screening, it is also possible to describe less suitable times that may impact on reliability, validity and sustainability, which is of particular relevance in the current context where efficiency and expediency are key.

The third objective of this particular pilot study was to ensure that the ambient noise levels did not exceed those suggested in literature. Measurement of the ambient noise levels in the screening environments also allowed the researcher to make the necessary adaptations to minimise these, which in turn contribute to the reliability and validity of hearing screening results. Establishment of noise levels in paediatric wards and clinics has implications for newborn and infant care. It has the potential to guide best practice by limiting noise exposure to newborns and infants through appropriate noise assessment and monitoring programmes (Neille, George & Khoza-Shangase, 2014).

### Research design

A descriptive, longitudinal, repeated measures, within-subjects design was employed. This research design was deemed appropriate as it made use of the same hearing screening measures on the same group of participants over time and allowed for all the necessary test–retest, between and within subject comparisons to be made (Schiavetti & Metz, 2006).

### Participants

A total number of 15 babies were discharged or being queried for discharge during the pilot study period. Of these 15 babies, 11 caregivers provided consent, three caregivers did not volunteer to participate and one baby was discharged before the initial hearing screening could be conducted. All 11 participants were booked for a follow-up hearing screening on the same day during their neonatal follow-up (6 weeks after discharge) to ensure that there were no false-negative screening results, and for assessing the appropriateness of implementing screening within the neonatal follow-up clinic should the neonate have missed in-ward screening as is often the case within the South African context. Neonates admitted to the NICU or high care wards (after birth) and transferred to ‘step down’ wards once medically stable, and for whom consent was obtained from the caregiver were included in the study. Neonates or infants who were previously discharged, returned home and were then readmitted to any of the wards were not enrolled in the study at the time of initial, in-hospital hearing screening.

### Procedures

Hearing screening was conducted using the AccuScreen OAE/AABR screener. Transient evoked otoacoustic emission (TEOAE) screening was conducted (frequency range: 1.5–4.5 kHz). This was followed by distortion product (DP) otoacoustic emission (DPOAE) screening that was conducted using the most comprehensive protocol on the machine with a 4/6 frequency *pass* criterion. AABR screening was conducted at the default level of 35 dBnHL using high forehead, cheek and nape of the neck electrode placements.

The average time required to screen each neonate or infant was established by recording the time taken to screen using all three screening measures and included the time taken to ensure appropriate probe fit and acceptable impedance. These times were available when downloading the data from the screening equipment and were recorded by subtracting the starting time from the time of completion. These recordings were then added and divided by the number of screening sessions in the study.

The most suitable time for the initial hearing screening in the wards was determined through field observation as well as through informal discussions with relevant personnel in the respective wards. The researcher engaged in collaborative discussions with consultant paediatricians and head nursing staff in the wards to establish the most suitable time for the initial hearing screening.

Ambient noise levels in the wards and the neonatal follow-up clinic were measured using a low-cost QUEST sound level meter, which is affordable, readily accessible, easy to use and has applications that include community and audiometric measurement or analysis. The sound level meter was placed at the caregiver’s bedside and the maximum noise level was measured and recorded for each screening session.

The initial hearing screening was conducted over a period of a week. Following completion of the initial hearing screening, results were explained to caregivers, and follow-up appointments were provided. Follow-up hearing screening was conducted approximately four to 6 weeks after the first phase in the morning, on days that coincided with neonatal follow-up clinic at the respective hospitals.

### Data analysis

Data were captured onto an excel spreadsheet and were analysed using descriptive statistics. Measures of central tendency such as the mean were used to summarise data. Data were also summarised using tabulated descriptions (Schiavetti & Metz, 2006).

## Ethical considerations

Ethical clearance was obtained from the University Medical Ethics Committee (M1211103) and permission was also obtained from relevant authorities at the research sites where the study was conducted. Informed consent was obtained from caregivers, and anonymity was ensured by assigning numbers to the completed data collection sheets. The home languages of the caregivers of participants were not recorded; however, all caregivers were able to speak and understand English and therefore did not require the use of an interpreter.

## Results and discussion

**FIGURE 1:**
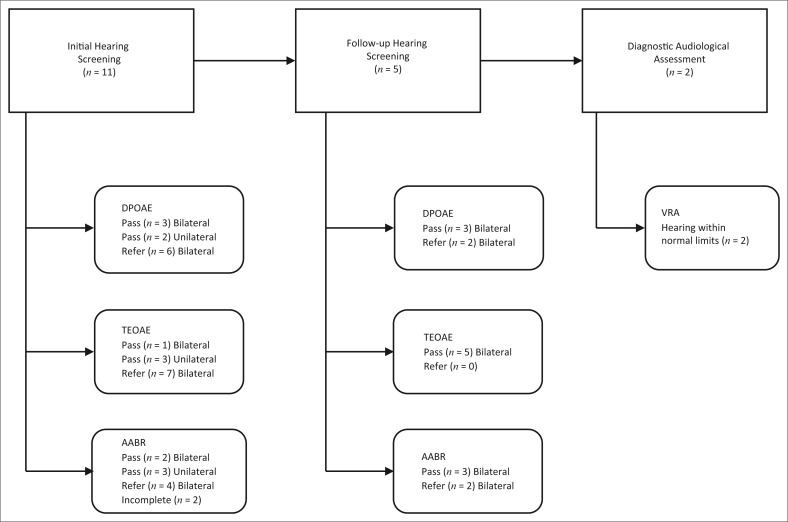
Outcomes of hearing screening protocol employed during data collection.

From the initial hearing screening, six participants presented with an overall bilateral *refer* result for DPOAE, four presented with a bilateral *refer* for AABR, and seven participants presented with an overall bilateral *refer* result for TEOAE. Three participants presented with a bilateral *pass* result for DPOAE, one participant presented with a bilateral *pass* result for TEOAE and a bilateral *pass* result for AABR was present in two participants. A unilateral *pass* result for DPOAE was present in two participants, with three participants presenting with a unilateral *pass* result for TEOAE and AABR (Table 1). AABR could not be completed on two participants as they were restless and difficult to calm (Figure 1). The high referral rate on one or more of the screening measures is consistent with literature that has also indicated a higher referral rate at the initial hearing screening in comparison with the repeat hearing screening (Chen *et al*., 2012; Colella-Santos, Hein, De Souza, Do Amaral & Casali, 2014). Referral rates have also been documented to decrease with an increase in age (Khoza-Shangase & Harbinson, 2015; Van Dyk, Swanepoel & Hall, 2015).

**TABLE 1:** Initial hearing screening result for each measure per participant.

Participant	Screening measure					
	DPOAE		TEOAE		AABR	
	Left	Right	Left	Right	Left	Right
1	Refer	Refer	Refer	Refer	Incomplete	Incomplete
2	Refer	Refer	Refer	Refer	Refer	Refer
3	Refer	Refer	Refer	Refer	Refer	Refer
4	Refer	Refer	Refer	Refer	Refer	Refer
5	Refer	Refer	Refer	Refer	Incomplete	Incomplete
6	Refer	Refer	Refer	Refer	Pass	Pass
7	Refer	Pass	Refer	Pass	Refer	Pass
8	Pass	Pass	Pass	Pass	Refer	Refer
9	Pass	Pass	Refer	Pass	Pass	Refer
10	Pass	Pass	Refer	Refer	Pass	Pass
11	Pass	Refer	Pass	Refer	Pass	Refer

DPOAE, distortion product otoacoustic emission; TEOAE, transient evoked OAEs; AABR, automated auditory brainstem response.

Of the 11 participants, six attended neonatal follow-up clinic with five participants having undergone a second hearing screening. The caregiver of the one participant had left the clinic following consultation with the paediatrician. Two of the five participants presented with *pass* results on all screening measures and were subsequently booked for behavioural audiometry at 6 months corrected age. Both participants attended the 6-month follow-up and presented with visual reinforcement audiometry results that were within normal limits. One participant underwent a third hearing screening due to incomplete results at the follow-up screening. *Pass* results were obtained for all screening measures, but there was no attendance at the 6-month follow-up. Two participants obtained bilateral *refer* results on both DPOAE and AABR measures and were subsequently booked for a diagnostic ABR. However, both participants did not attend this follow-up appointment. These findings highlight the poor follow-up return rate in NHS programmes, which has been a widely reported challenge in both developing and developed contexts. The poor follow-up return rate in the current pilot study is consistent with findings from a community-based screening programme in Nigeria where more than half of the participants did not return for the second screening even though services were offered free of charge (Olusanya & Akinyemi, 2009). These findings highlight the need to explore contextual factors (other than cost) that influence follow-up return rate, such as the influence of socio-economic status, cultural and linguistic factors within the South African context.

### Description of participants in pilot study

The pilot study comprised 11 participants. Two of the caregivers of participants were Zimbabwean, and the remaining nine were South African. With regard to ethnicity, all 11 participants were Black African, with nine of the 11 participants being female and two being male. The ethnic profile of participants is reflective of the national estimates of the general South African population as well as the differences in access to, and use of public versus private healthcare facilities. Black Africans constitute approximately 80% of the total population in South Africa and predominantly make use of public sector health services (Statistics South Africa, 2013a, 2013b, 2014). The average gestational age was 30 weeks, with six participants being classified as very low birth weight, one as low birth weight and two as extremely low birth weight. One participant had a normal birth weight of 2880 g, and for another participant the birth weight was not recorded in the file (Table 2).

**TABLE 2:** Description of participants.

Participant	Gender	Gestational age (weeks)	Birth weight (grams)	Length of hospital stay (days)
1	Female	32	Unknown	10
2	Female	32	1480	17
3	Female	30	1440	15
4	Male	27	1000	34
5	Female	40	2880	3
6	Female	28	1390	24
7	Female	31	1500	10
8	Female	30	1460	21
9	Male	27	820	26
10	Female	29	1240	29
11	Female	27	730	34

None of the participants had been admitted to the NICU, but all had a prolonged hospital stay. The mean stay in high care was 7 days, with a longer stay in the Kangaroo Mother Care (KMC) ward for an average of 14 days. Three of the 11 participants underwent phototherapy due to neonatal jaundice. With regard to retroviral disease (RVD) exposure, two participants were RVD exposed, seven were unexposed and the RVD status of two participants was unknown. Medication considered to be ototoxic was administered to eight of the 11 participants during their hospital stay. These ototoxic drugs consisted of gentamycin, amikacin and vancomycin, with gentamycin having been the most frequently administered drug in seven of the participants.

### Time required per screening measure for each neonate

The average time to complete TEOAE screening was one minute per ear. DPOAE screening time was slightly longer with the average time of two minutes per ear, and AABR screening time was similar to DPOAE with an average time of two minutes per ear (one minute, 55 seconds). The average time taken to complete the hearing screening with all three measures was 18.4 minutes as this included obtaining good probe fit and ensuring that participants were calm when conducting each screening measure (Table 3).

**TABLE 3:** Time taken to complete each of the screening measures at the initial screening in minutes and seconds.

Participant	DPOAE		TEOAE		AABR	
	Left ear	Right ear	Left ear	Right ear	Left ear	Right ear
1	01:36	01:55	01:16	00:52	Incomplete	Incomplete
2	00:28	02:38	01:11	01:09	02:12	-
3	02:16	02:01	01:15	01:11	02:40	-
4	02:46	02:15	01:16	00:13	02:09	-
5	03:08	03:13	01:19	01:22	Incomplete	Incomplete
6	01:43	02:22	01:20	01:02	01:29	-
7	02:22	01:45	00:56	01:30	02:05	-
8	01:03	01:52	00:15	00:12	02:48	-
9	01:01	02:01	01:23	00:23	02:28	-
10	01:38	01:16	01:10	01:23	00:49	-
11	01:35	03:32	00:17	01:26	01:18	-

DPOAE, distortion product otoacoustic emission; TEOAE, transient evoked OAEs; AABR, automated auditory brainstem response.

The researcher observed three factors that influenced the time taken to complete each of the screening measures. The first factor was the state of the newborn or infant, the second factor was the signal-to-noise ratio for OAE screening and the third factor was the EEG for the AABR. Preferably, the newborn or infant should be resting quietly in a bassinette or crib, and if needed may be held (ASHA, 2015). The best results were obtained for babies who were awake but calm, or for sleeping newborns or infants with a good signal-to-noise ratio on OAE screening and a good EEG, as these factors resulted in a shorter test time. With regard to OAEs, particularly DPOAEs, the larger the DP to noise ratio, the quicker the criteria for the DP are met, which in turn results in a shorter test time (Hall, 2000) and a good EEG as an influencing factor is consistent with reports that the newborn’s state of consciousness influences the time to complete the assessment (Sena-Yoshinaga, Almeida, Côrtes-Andrade & Lewis, 2014). These findings have important implications for screening programmes as they indicate both the time requirements as well as possible influencing factors, which, if managed, would lead to the success of a screening programme, especially in an under-resourced environment. Current findings are consistent with those reported in a number of studies that have reported AABR test time to be longer than when assessing using OAEs. This has been based on the premise that additional time is required for electrode placement (Berg, Prieve, Serpanos & Wheaton, 2011; Meier, Narabyashi, Probst & Schmuziger, 2004; Norton *et al*., 2000). However, longer test time for AABR has also been noted in the study by Van Dyk and colleagues that employed TEOAE and the MB11 BERAphone, which does not require placement of disposable electrodes (Van Dyk *et al*., 2015). Contrary findings have been reported in the study by Sena-Yoshinago and colleagues who reported an AABR screening time close to that of TEOAE, with a mean assessment time of 32.9 seconds (Sena-Yoshinaga *et al*. 2014). These authors further concluded a shorter test time for newborns that were in stage 1 of consciousness (deep sleep, no movement, regular breathing).

### Most suitable days and times for screening

From the discussions with nursing staff, it appears as if the afternoons were better suited for initial hearing screening, as ward rounds were usually completed. It was easier to identify babies who were going to be discharged or being queried for discharge during this time. The noise levels were significantly less because student training in academic hospitals usually occurs on ward rounds during the morning. The most suitable time for screening appeared to be between feeding times when babies were generally comfortable, satisfied and sleeping. These are ideal conditions for hearing screening where objective measures are utilised and sleeping is the preferred neonatal state of arousal. Babies were reportedly weighed on Tuesday and Friday mornings at the one hospital, unless they weighed < 1500 g, in which case they got weighed daily. Based on the initial aims of the pilot study, these were therefore identified as the most suitable days for hearing screening at this research site. To prevent babies from being omitted from screening on days that the researcher was not in the hospital, it seemed best to screen babies on the days they got weighed (e.g. Tuesdays and Fridays in this case). Babies at the second hospital were weighed everyday but Mondays and Thursdays were recommended days by paediatricians for the initial hearing screening from 11:00 onward following completion of ward rounds. These recommended differences amongst hospitals highlight the need for audiologists to explore the site-specific, routine care offered at the respective hospitals prior to the implementation of NHS.

Caregivers were usually present at all times in two of the wards at the first hospital and were always present in the KMC ward at the second hospital. Difficulties were sometimes experienced in the high care wards at both hospitals due to a lack of accommodation for mothers in these wards. Some of these mothers had to be admitted to another ward for medical care whilst their babies were cared for by nursing staff in these wards.

### Ambient noise levels

Ambient noise levels should also be taken into consideration when deciding on the most appropriate time to conduct hearing screening, especially in the wards within a hospital-based context. The noise levels not only have an effect on the screening time, but also on the sensitivity and specificity of the objective screening measures employed within an NHS programme (Salina, Abdullah, Mukari & Azmi, 2010). Although Khoza-Shangase and Harbinson (2015) have suggested that in order for OAE measures to be reliable, ambient noise levels should not exceed 50 dBA to 55 dBA of noise; current sound level readings ranged between 50 dBA and 70 dBA in the KMC wards (Table 4), and screening in the current sample was deemed possible.

**TABLE 4:** Sound level recordings within the screening environments at the first hospital.

dBA: In-patient initial screening	dBA: Out-patient follow-up screening
59.5	-
64.3	56.0
63.0	-
50.0	-
70.0	-
64.0	40.0
56.0	56.0
52.9	57.0
58.8	-
58.3	58.5
58.9	-

dBA, Sound level measurement.

The average sound level was 59.6 dBA, making screening in these wards possible. Some authors have reported that accurate OAE screening results are obtained when sound levels do not exceed 65 dBA – 68 dBA (Olusanya, 2010; Salina *et al*., 2010). Screening was not conducted in the high care ward as there were only two babies during the pilot study that were being discharged. The one caregiver left the hospital prior to having her baby’s hearing screened, and the second baby was transferred to a different ward where the screening was then done. However, this baby was full term with a normal birth weight, and the caregiver was being referred to a nearby clinic for follow-up after discharge. Sound level readings ranged between 48 dBA and 60 dBA in the KMC ward at the second hospital. The high care ward at this hospital was not a suitable environment for hearing screening due to multiple cribs per cubicle, noise generated by alarms on incubators and a high volume of medical and nursing staff conducting routine care and training of medical students during the course of the day. Similar noise sources have been reported in a study conducted at private and public sector hospitals in South Africa (Neille *et al*., 2014). It was therefore decided that babies in cribs would be tested in the last empty cubicle used for storage in the high care ward or an empty cubicle in the KMC ward, where ambient noise levels could be controlled.

The average sound level in the screening environment at the neonatal follow-up clinic at the first hospital was 57.25 dBA. These sound levels were lower at the second hospital and ranged between 40 dBA and 50 dBA as the follow-up hearing screening was conducted in the audiology department due to a lack of available consulting rooms in the clinic. The audiology department was a short distance away from the clinic, which allowed for fairly easy access to the participants.

### Limitations

It is acknowledged that the data used to describe the context are site-specific and need to be explored on a larger scale for similar contexts. It is further acknowledged that the limited sample size in the current pilot study prevented the generalisation of findings. These findings need to be considered when planning for larger scale NHS studies or when planning a hospital-based NHS programme. Larger scale studies on the implementation of a NHS programmes are necessary in the South African context.

## Conclusion

Findings suggest that NHS can be conducted in public sector hospitals in South Africa, provided that test time is considered in addition to sensitivity and specificity when deciding on the screening protocol to be adopted. These factors have even more relevance within the South African context where limited resources are an important consideration. Furthermore, high sensitivity and specificity of the adopted screening protocol can possibly facilitate use of non-audiologists in screening programmes where minimum standards of training screeners have been adhered to. Implementation of an NHS programme over a 12-month period, with consideration of test time, ambient noise levels, test sensitivity and specificity, may provide more information regarding the feasibility in a hospital setting. It is recommended that the impact of ambient noise levels on the time taken to complete screening as well as the screening outcomes be further explored in future studies. Current findings seem to indicate ambient noise levels at the edge of the desired maximum limits within the screening contexts; therefore, careful monitoring of noise levels as part of any screening protocol is highlighted as important. Careful attention to these factors would improve the efficacy of neonatal screening programmes within the high demand to capacity ratio South African audiologists function under.
